# Characterization of Reddit Posts About Xylazine-Associated Wounds: Qualitative Study

**DOI:** 10.2196/70329

**Published:** 2025-09-12

**Authors:** Anthony Spadaro, JaMor Hairston, Sahithi Lakamana, Rachel Wightman, Jennifer Love, Jeanmarie Perrone, Abeed Sarker

**Affiliations:** 1 Department of Emergency Medicine, Rutgers New Jersey Medical School, 140 Bergen St, Newark, NJ, 07103, United States, 1 973-972-2977; 2 Department of Biomedical Informatics, Emory University, Atlanta, GA, United States; 3 Department of Emergency Medicine, Brown University, Providence, RI, United States; 4 Department of Emergency Medicine, Icahn School of Medicine at Mount Sinai, New York, NY, United States; 5 Department of Emergency Medicine, University of Pennsylvania, Philadelphia, PA, United States

**Keywords:** xylazine, social media, substance-related disorders, opioid-related disorders, wounds

## Abstract

**Background:**

Xylazine has been associated with skin wounds. The rising prevalence of xylazine and its debated role in wound causation have sparked concerns among public health professionals, medical experts, and people who use drugs.

**Objective:**

This study used a qualitative evaluation of Reddit posts to understand the experiences of people who use drugs concerning xylazine-associated wounds.

**Methods:**

This study explored xylazine discussions on Reddit. Data were collected from 930+ drug-related subreddits via the PRAW Python application programming interface, and natural language processing methods were employed to identify posts that mentioned xylazine and wound-related keywords. Retrieved posts were manually coded for thematic analysis, and a term frequency–inverse document frequency analysis was performed per theme to obtain additional insights.

**Results:**

The manual classification of 286 posts revealed predominant themes related to the pathophysiology of xylazine, wound locations on the body, and management strategies. The 3 most frequent xylazine wound-related themes were “Mechanisms of xylazine-associated wounds” (84 posts, 29.4%), “Geographic region” (67, 23.4%), and “Location of wounds on the body” (56, 19.6%). The analysis showed xylazine’s presence in the discussions among Reddit’s drug-using communities, with a notable focus on wound management and geographic trends. The term frequency–inverse document frequency analysis revealed prominent lexical markers within each theme.

**Conclusions:**

The findings suggest that social media platforms such as Reddit can serve as valuable resources for understanding emerging health issues such as xylazine-associated wounds. The study’s findings highlight patterns of use, the characteristics of wounds on people who use drugs, and discussions about wound management. This study adds to a growing body of literature using social media to understand the consequences of emerging drugs on human health.

## Introduction

Xylazine is an α -adrenergic agonist that is increasingly prevalent in the unregulated opioid supply [1]. In the northeast United States, xylazine may be present in more than 90% of the fentanyl supply in some localities [2]. As the prevalence of xylazine has increased, there has been growing concern voiced by people who use drugs, the media, and medical providers that xylazine use is associated with skin wounds [3,4]. The etiology of wounds associated with xylazine exposure, the relationship to route of drug use, the location on the body where the wounds develop, and the optimal treatment of wounds are not known [1-3]. Theories proposed include vasoconstrictive effects, tissue hypoxia, cytotoxicity, impaired glucose control, prolonged sedation, pressure-related injury, and other factors associated with drug use practices, including access to sterile supplies, proper nutrition, and clean water [5,6]. Despite these unknowns, there is significant concern from public health and government regulatory agencies about the association between xylazine and skin wound development [6].

Xylazine-associated wounds are extensively discussed among people who use drugs, including on social media [7]. Reddit is a popular social network that allows for anonymous posting by subscribers (redditors), with more than 1.2 billion monthly active subscribers [8]. Because of its anonymity, Reddit has become a forum for people to discuss sensitive or stigmatized topics such as drug use [9]. For these reasons, social media in general, and Reddit specifically, has been analyzed to understand various aspects of drug use [7,10]. Various social media sites such as Reddit, X, or TikTok may be used to study attitudes toward drugs; however, each site attracts different demographics, may restrict discussion of drug-related topics, and has different rules for researchers to extract data [10]. Reddit has been used for pharmacovigilance of emerging substances, to explore adverse effects from prescribed medications in stigmatized communities, and to explore the general public’s perceptions of drug-related topics [7,11,12]. Others have used Reddit to study perceptions of xylazine in general and noted concerns about wounds as an adverse effect, although this study did not focus specifically on issues related to xylazine-associated wounds [13].

In prior work, we leveraged natural language processing (NLP) to identify social media posts on drug-related topics of interest to medical toxicology and addiction medicine researchers and used these social media posts to identify potential adverse effects of xylazine use [7]. For this study, we sought to specifically explore an adverse effect that was identified in our prior work, xylazine-associated wounds. Our primary aim was to perform a thematic analysis of Reddit posts specifically related to xylazine and wounds in order to identify associated factors and salient issues xylazine-associated wounds may be causing for people who use drugs.

## Methods

### Ethical Considerations

This study was approved by Emory University Institutional Review Board (number STUDY00002458). We combined NLP and expert-driven qualitative analysis to thematically characterize a set of Reddit posts mentioning xylazine and wounds. Posts were not modified from their original form in order to not alter their meaning. All data were publicly available and anonymous at the time of collection. Usernames were not included in the data analysis to further protect the privacy and anonymity of the Reddit users. The authors had no contact with individual Reddit users and no compensation was provided to any Reddit users.

### Data Collection

We collected data from 961 drug-related subreddits (Appendix 1 in Multimedia Appendix 1) via the PRAW (Python Reddit API Wrapper) application programming interface (API) (version 7.7.1; GitHub). We used Python (version 3.12; Python Software Foundation) for PRAW and for filtering the data. Data filtering was conducted using the Natural Language Toolkit for tokenization and stop word removal, and regular expressions (via Python’s remodule) to identify and exclude noninformative or low-quality posts [7]. The API enables the collection of publicly available data from chosen subreddits in a secure manner following authorization. Posts that are removed by moderators or the original posters are not available via the API. No subreddit that had protected data and required joining prior to viewing data was included in this study. First, we filtered all the collected data using the keywords “xylazine,” “tranq,” and other possible lexical variants, including street names and common phonetic and typographic misspellings (Appendix 2 in Multimedia Appendix 1) [14]. Then, we filtered the data using a predetermined set of keywords (Appendix 2 in Multimedia Appendix 1) potentially related to wounds (eg, mentions of wounds, necrosis, and skin ulcers). Thus, posts mentioning xylazine and potentially wound-related information were included for further thematic analysis.

### Data Analyses

A sample of 15 posts identified by NLP as having content related to xylazine and wounds was reviewed by 2 authors with expertise in toxicology (ASp and JP) to identify relevant categories (codes) into which the posts could be classified [7]. Codes were developed using a content analysis approach [15]. ASp and JP prepared a guidebook to drive the coding of the social media posts (Table S1 in Multimedia Appendix 1). An initial randomized set of 50 posts was independently assigned codes by 2 authors (ASp and JH). The larger author group reviewed the coding assignments, and the codes were iteratively defined through consensus until agreement was reached regarding the definitions of the codes and their adequacy in describing the themes in the posts. This process ensured that codes that were too inclusive or poorly defined could be eliminated, and additional codes could be created. This iteration generated 13 primary codes, including 2 codes: “non-relevant post about xylazine” and “not about xylazine at all” for posts that lack relevance to human xylazine use (eg, posts purely about veterinary use of xylazine) and posts that lack relevance to xylazine completely, respectively. Each code represented a potential theme that could be present in a post, and each post could be assigned multiple codes if the post contained multiple themes [15]. Table 1 shows the 13 codes and their definitions.

**Table 1.** Themes from posts extracted by natural language processing with example quotes, distribution, and indicative n-grams detected via term frequency–inverse document frequency.

Theme	Example quotes	Number of posts coded	Percentage of posts with theme (out of 286)	Illustrative top TF-IDF^a^ terms and values
Hypothesized mechanisms of xylazine-associated wounds	“it’s a Vasoconstrictor and Will Greatly Reduce the Ability of Oxygen-Rich Blood to Get Out into All the Small Veins. If You Repeatedly Inject into a Single Site, You’re Counting on Your Circulatory System to Repair the Damage and Catch Any Bacterial Infection.”	84	29.4	Top terms include xylazine (184.91) itself, krokodil (29.38) for comparison, xylazine induced (19.09) for direct effect, skin necrosis (15.22), skin ulceration (16.70), and references to pmc articles (10.45).
Geographic region	“This shit is RAVAGING South Jersey.”	67	23.4	Prominent terms include philly (21.74), harm reduction (11.24), care supplies (11.17), and https www (10.16) referring to web-based sources. Xylazine skin necrosis (20.65) is noted as a health consequence by locale.
Locations of wounds on the body	“It ate my skin on both arms. They looked horrible, talking down the bone.”	56	19.5	Top terms for this theme include xylazine (122.35) itself, alongside skin (52.44), oxygenation (20.99), skin ulceration (16.70), and skin necrosis (13.04), indicating the severe nature of wounds.
Management of wounds	“Other than OP’s suggestion to have good hygiene (which I think meant using clean supplies & cleaning IV sites) I personally use saline nasal spray to clean my nose out about 15‐20 min after snorting.”	34	11.8	Illustrative terms include xylazine (99.36), oxygenation (20.99) as critical for healing, skin ulceration (16.70), impaired healing wounds (7.16), and whole wrap (12.00) possibly for bandaging.
Posts about specific xylazine withdrawal symptoms	“I’m wondering if the withdrawal anxiety (I don’t rly get any other withdrawal symptoms it’s like the methadone covers any sickness and other parts of withdrawal except for the anxiety which makes me think the rebound is not from the fent but from the cut…possibly xylazine) so yeah I was saying I wonder if the “withdrawal” or rebound anxiety is rly from sniffing a shit ton of xylazine.”	29	10.1	Examples reflecting withdrawal symptoms include withdrawals (14.13), organs hurting (6.97), zombifying bodies (7.16), and every hour (10.49). Tranq dope (17.00) indicates polysubstance context.
Stigma related to xylazine wounds	“Tranq will actually keep your wounds from healing, and they are calling it a zombie drug.”	23	8.0	Examples reflecting stigma include terms such as flesh eating (11.93), skin rotting (9.37) for graphic descriptions, zombie (12.65) for dehumanization, dirty (11.17) for uncleanliness, and opioid deaths (9.54) for links to crises.
Other drug use habits	“Makes me think it’s the common analog in benzos called Etizolam.”	20	6.9	Top terms illustrate polysubstance use such as xylazine (46.92) with morphine (24.38) and fent (fentanyl). Phrases such as fent mixed xylazine (4.20), every hour (10.49) for frequency, and methadone clinic (5.58) are also key.
Xylazine use habits	“I got lucky with my IV use, never got hep or anything but I did get a bad infection from a muscle injection.”	19	6.6	Illustrative top terms include nose (18.48) for intranasal use, xylazine injectable (10.0 and 8.38) for injection, every hours (10.49) for frequency, fent (11.0) for polysubstance use, and skin (15.64) for side effects.
Posts about MOUD^b^	“Now I have methadone in me yet it isn’t making a difference now that’s probably cause from using on top of the done I jacked my tolerance so high I no longer feel the methadone at all it’s mine as we’ll be water I need to increase my dose, but it seems like something else is going on.”	19	6.6	Examples include xylazine (65.32) caused with MOUDs such as methadone (17.39), phrases such as suboxone kill cravings (4.77), terms such as fentanyl (21.16), and community queries such as quick question anyone (4.22).
Non-MOUD management of withdrawal	“Yes clonidine is the best drug I’d say for coming off tranq.”	11	3.8	Examples include xylazine (19.32) and benzos (7.73) for self-medication, 3-mg clonidine (2.81), terms such as noradrenaline new (3.48), and indicators of injection risks such as iv warned (2.81).
Ability to get into rehabilitation clinic or addiction treatment	“I can’t get wounds healed & rehabs won’t take me with open festering wounds.”	10	3.5	Key terms include rehab (16.70), xylazine (11.04) as a complicating factor, phrases such as hospitals around area (4.77), save someone’s life (4.77) for motivation, and insurance xylazine imagine (3.48) as a barrier.
Nonrelevant posts about xylazine	“I run a wildlife hospital and we use xylazine in a lot of our cases. Because it’s not a scheduled drug it’s easy for us to keep on hand for euthanasia, sedation for fractious animals needing wound care, exams, etc.”	37	12.9	N/A^c^
Not about xylazine at all	“If I’m remembering right wouldn’t him jumping into a gross jungle river soon after losing it cause some serious infection, especially in the 60 s?”	70	24.4	N/A

a TF-IDF: term frequency–inverse document frequency.

b MOUD: medications for opioid use disorder.

c N/A: not applicable.

Using the guidebook, author JH manually coded the remaining posts. All the coded posts were reviewed by authors ASp and JP, and any disagreement about the assigned codes was discussed as a group and resolved by consensus. Following the thematic categorization, word n-gram (n=1‐3)–level term frequency–inverse document frequency (TF-IDF), where each document included all the posts within a specific theme, was computed to identify n-grams uniquely indicative of each theme. An n-gram is a sequence of n adjacent symbols in a particular order—in this context, a contiguous sequence of words. The top n-grams for each theme were processed with a large language model (LLM) (Google Gemini 2.5) for summarized explanation and interpretation. The 2 noninformative themes were excluded from this analysis. The TF-IDF analysis of the contents associated with each theme and their LLM-assisted explanations revealed some key topics associated with each theme.

## Results

**Figure 1.**
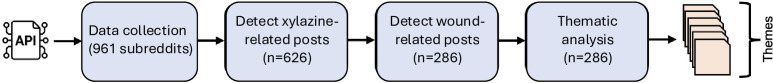
Flow diagram of data collection to thematic analysis. API: application programming interface.

We retrieved 5373 posts from 961 subreddits from January 2019 to March 2023. A total of 626 posts were detected to potentially mention xylazine. Within these posts, 286 posts were detected via NLP to contain potentially wound-related keywords, and all of these posts were manually reviewed for thematic analysis (Figure 1). Of these 286 posts, 37 (12.9%) of posts were about xylazine but determined to be about nonhuman use, such as veterinary use. An additional 70 of the 286 posts (24.4%) were determined to be not about xylazine at all and were either incorrectly selected by NLP or were nonrelevant comments on posts about xylazine and wounds. In total, 179 of the 286 (62.6%) posts extracted by NLP were about xylazine and wounds. The posts described several important aspects of the experiences people shared on Reddit about xylazine-associated wounds.

The 3 most frequent xylazine wound-related themes were “Hypothesized mechanisms of xylazine-associated wounds” (84/286 posts, 29.4%), “Geographic region” (67/286, 23.4%), and “Location of wounds on the body*”* (56/286, 19.6%). Table 1 shows the frequencies of posts for 13 xylazine wound-related themes, along with representative examples. Less prominent themes were “Management of wounds*”* (34/286, 11.8%), “Posts about specific xylazine withdrawal symptoms*”* (29/286, 10.1%), and “Stigma related to xylazine wounds*”* (23/286, 8.0%), which contained important information about managing wounds, their impacts, stigma, and withdrawal.

Posts described how the redditors were using xylazine and speculated on the relationship between how xylazine was being used and the development of wounds. For example, “People are getting wounds on their noses and mouths. Sniffing and smoking is giving people sores.*”* Other posts discussed specific mechanisms and the pathophysiology of xylazine and how it may cause wounds, for example, “It causes severe vasoconstriction which is why it leads to wounds.*”* Some sought help in managing wounds and described their wounds, for example, “How do you treat these skin wounds? I have them on my chest, arms, and legs. How do I know if I need to see a doctor? Any info what to do would be great.” The discussions of the pathophysiology and mechanisms of xylazine were often connected to discussions on the locations of the wounds and how xylazine was being used, seemingly to provide explanations for why certain wounds were developing. For example, “Apparently it adds longevity to the high. However the side effect is that it’s much harder on the blood vessels. As blood vessels break down, infection risk skyrockets. Users are forced to find new veins more frequently, injection sites don’t heal well and this is the result. Infection and destroyed blood vessels causes strain to organs, and the extremities are starved for blood.”

Some of the posts expressed that having xylazine-associated wounds made getting addiction treatment more difficult; for example, one post described someone’s experience trying to get into addiction treatment: “She wants to, but they won’t take her into rehab with the wound and without rehab she’ll likely keep injecting.*”* Many of the posts about wounds also discussed issues with attempts at stopping the use of xylazine and unregulated opioids. Posts described both the attempts at quitting “cold turkey” and using medications for opioid use disorder (MOUD), “Was hooked on Philly tranq bad from Kensington for years. It ate my skin on both arms. They looked horrible, talking down to the bone. The withdrawals would be so bad. Nonstop throwing up, literally every couple mins. Even when nothing is left, you’ll just puke bile or dry heave. It’s terrible. I was stuck on it for years. Was on 200 mgs of methadone on top of it which did absolutely nothing.*”* Posts that commented on these themes of getting into addiction treatment, complicated withdrawal treatment, and xylazine wounds were sometimes connected. These posts would describe how xylazine made withdrawal more complex to manage and that made wounds more difficult to treat. For example, “There isn’t a lot of good hospitals around that area that actually seem to know what they are doing for xylazine/fentanyl withdrawal and that one I mentioned does an excellent job and if some one reads it, and can save someone’s life I don’t see the harm in posting that. My fiancé died last year because we both thought the hospitals around that area would not know how to treat her withdrawal for an infection. And the infection spread into her blood and she died.”

Several posts disclosed the geographic location of where the poster was, where they were buying drugs, or where they were seeing people with wounds. Many of the posts used stigmatizing language to describe wounds and the people who get them, “I do heroin regularly and never had any issues. These people are dirty junkies who don’t take care of themselves and won’t even swab their skin with an alcohol pad but it’s definitely not a combination of unsanitary conditions and dirty drugs full of flesh eating bacteria that can be filtered out with cheap micron filters.*”* Many of the posts also referred to xylazine as the “zombie-drug” and that people who used it were “zombies.” Many posts with stigmatizing language were directed toward others rather than the person making the post. As posts were anonymous, it was not known in all instances whether the redditor making the post was a person who uses drugs. However, some posts with stigmatizing language did seem to come from health care providers, and some of the stigma seemed to be coming from the non–people who use drugs community. For example, one post from a health care provider described their first encounter with a patient who used xylazine, “I met my first patient with tranq wounds yesterday. Both their legs look absolutely awful, and the smell is just unspeakable.”

The TF-IDF analysis revealed some key topics associated with each theme, such as “Philly” as a prominently mentioned location, “wraps” or bandages for wound management, and graphic descriptions (eg, “flesh eating” and “zombie”) expressing stigma associated with xylazine-inflicted wounds. Further examples and explanations are shown in Table 1.

## Discussion

### Principal Findings

This study uses social media data and NLP to explore a critical clinical complication of the emerging drug xylazine. In this study, social media posts described purported mechanisms behind toxicity from xylazine, experiences with xylazine wounds including how people managed wounds, and stigma related to xylazine use. While the association between xylazine and the development of distinct wounds has been described in case reports and the media, there is limited understanding of the pathophysiology, the optimal management of these wounds, and individual experiences with xylazine-associated wounds [3-6,16-18]. There have been many postulated mechanisms by which xylazine could cause wounds. Xylazine may cause vasoconstriction through its α-adrenergic agonist effect, which could cause tissue hypoxia and injury [6]. Xylazine may also have systemic cytotoxic or hypoxic effects, which have been proposed as a possible explanation for wounds distant from the site of drug use [6]. There are reports of people developing wounds at sites where they do not inject and in those who report only intranasal use [3]. Some experts are skeptical about whether these wounds are a distinct clinical entity separate from skin and soft tissue infections that can occur with intravenous drug use [5]. The results of this study show that people self-report developing wounds after using xylazine. Many of the posts commented on the development of wounds in sites where they do not inject and in association with intranasal use. This has been previously reported, for example, at a wound care clinic in Philadelphia, patients reported developing wounds on their extremities at sites where they do not inject and in those who reported only intranasal use [3]. The development of wounds in the nose and oropharynx is not well described but may be clinically relevant, and it is notable that it is mentioned on Reddit before it is described in the medical literature. These findings support that xylazine-associated wounds can develop without intravenous use. Additionally, many posts speculated on the pathophysiology of xylazine and how it might lead to wound development. While this does not prove an association between xylazine and wound development, it emphasizes the concern about xylazine-associated wounds among Reddit users who are posting on drug-related subreddits and the need for more research into causation.

Many of the posts also used stigmatizing language and employed terms such as “zombie” to discuss the wounds and the people who have them. While this language may reflect the language used in popular media around xylazine use, it is of concern that xylazine-associated wounds may confer additional stigma on a population that already faces a significant amount of stigma [16-20]. Although the posts were anonymous, people occasionally self-disclosed drug use, and there were some notable examples of stigmatizing language used by people who use drugs without xylazine-associated wounds toward people who use drugs with xylazine-associated wounds. Stigma toward people who use drugs with xylazine-associated wounds from popular media and health care institutions has been described, but less is known about stigma within the people who use drugs community around xylazine wounds [20].

Published experiences from low-barrier wound clinics have reported that patients with xylazine-associated wounds face significant structural barriers and fear of withdrawal that may prevent them from getting adequate wound care [4]. Several of the posts discussed withdrawal symptoms that were attributed to xylazine and inadequate relief of their withdrawal symptoms with MOUD. Withdrawal symptoms and issues with MOUD were often brought up in the context of wanting to quit using xylazine so that wounds would heal. Some posts also discussed difficulty with getting into inpatient detox, residential, and rehabilitation treatment facilities due to the presence of wounds. These posts highlight that xylazine may complicate the treatment of opioid use disorder if people are having difficulty getting into treatment programs or staying on MOUD [3,4].

### Limitations

There are several limitations to this study. The posts were self-reported experiences without confirmation that xylazine was present in the drugs the redditors were reportedly using. Redditors who experience more negative effects of drug use may be more likely to post about it. Additionally, this analysis was limited to publicly available posts, and thus private posts were not included, and the posts analyzed may not be representative of everyone’s experiences. Individuals who post on Reddit may not be representative of the larger community of people who use drugs, particularly those without phone or internet access. Another limitation is that only keyword-matched posts were included in the thematic analyses; thus, potentially relevant information in posts not containing the required keywords was excluded. The filtering, however, was a necessary step since it is not feasible to review the large volume of content generated on Reddit manually. Future work could expand on the relatively small number of posts analyzed here by looking at other social media sites, which may have different user bases and find a broader range of themes. As the posts were anonymous, we do not have demographic information on the redditors to know in which ways they might be different from other populations. More research is needed to understand the real-world impact of these issues being discussed on social media. Future studies using social media analysis should include exploring other related emerging drugs such as medetomidine, nitazenes, and tianeptine. Future work will include exploring machine learning methodologies such as LLMs to automate the classification performed in this study. Automating this process will increase the feasibility of scaling it for much larger datasets of social media posts and allow for more rapid analysis of studies on emerging drugs. This study has been informed by the clinical experience and expertise of some of the authors; however, there may be delays between when a novel drug emerges and when a clinician may encounter it in a health care setting. Future work should explore the ability to identify emerging drugs of abuse being discussed on social media before expert clinicians even begin to encounter them in the health care setting, which could allow for health care systems to proactively prepare for emerging drugs.

### Conclusions

Reddit posts revealed discussion behind the pathophysiology of xylazine-associated wounds and discussed the impact of wounds on treatment and service access. As the unregulated drug market changes and new drugs emerge, social media may continue to be a valuable resource to study adverse effects from novel drugs and their impact on the people who use them.

## Supplementary material

10.2196/70329 Multimedia Appendix 1 List of subreddits used, lexical variants searched, and definition of themes.

## Abbreviations

API: application programming interface
LLM: large language model
MOUD: medications for opioid use disorder
NLP: natural language processing
PWUD: people who use drugs

## References

[1] Friedman J, Montero F, Bourgois P (2022). Xylazine spreads across the US: a growing component of the increasingly synthetic and polysubstance overdose crisis. Drug Alcohol Depend.

[2] Zhu DT (2023). Public health impact and harm reduction implications of xylazine-involved overdoses: a narrative review. Harm Reduct J.

[3] McFadden R, Wallace-Keeshen S, Petrillo Straub K (2024). Xylazine-associated wounds: clinical experience from a low-barrier wound care clinic in Philadelphia. J Addict Med.

[4] Reed MK, Imperato NS, Bowles JM, Salcedo VJ, Guth A, Rising KL (2022). Perspectives of people in Philadelphia who use fentanyl/heroin adulterated with the animal tranquilizer xylazine; making a case for xylazine test strips. Drug Alcohol Depend Rep.

[5] Hoffman RS (2023). Closing the xylazine knowledge gap. Clin Toxicol (Phila).

[6] D’Orazio J, Nelson L, Perrone J, Wightman R, Haroz R (2023). Xylazine adulteration of the heroin-fentanyl drug supply : a narrative review. Ann Intern Med.

[7] Spadaro A, O’Connor K, Lakamana S (2023). Self-reported xylazine experiences: a mixed-methods study of Reddit subscribers. J Addict Med.

[8] Dean B (2024). Reddit user and growth stats. Backlinko.

[9] Chi Y, Chen H yu (2023). Investigating substance use via Reddit: systematic scoping review. J Med Internet Res.

[10] Carpenter KA, Nguyen AT, Smith DA (2025). Which social media platforms facilitate monitoring the opioid crisis?. PLOS Digit Health.

[11] Zhu J, Zhang X, Jin R, Jiang H, Kenne DR (2025). Probing public perceptions of antidepressants on social media: mixed methods study. JMIR Form Res.

[12] Gilbert MK, Daughton AR, Chilcoat HD, Laffont CM, Strafford S, DeVeaugh-Geiss AM (2025). Social listening for patient experiences with stopping extended-release buprenorphine: content analysis of Reddit messages. J Med Internet Res.

[13] Heidari O, Sugarman OK, Winiker AK (2025). Personal experiences with xylazine and behavior change: a qualitative content analysis of Reddit posts. J Addict Med.

[14] Sarker A (2021). LexExp: a system for automatically expanding concept lexicons for noisy biomedical texts. Bioinformatics.

[15] Vaismoradi M, Turunen H, Bondas T (2013). Content analysis and thematic analysis: implications for conducting a qualitative descriptive study. Nurs Health Sci.

[16] Hoffman J (2023). Tranq dope: animal sedative mixed with fentanyl brings fresh horror to U.S. drug zones. The New York Times.

[17] Sue KL, Hawk K (2024). Clinical considerations for the management of xylazine overdoses and xylazine-related wounds. Addiction.

[18] Jawa R, Ismail S, Shang M (2024). Drug use practices and wound care experiences in the age of xylazine adulteration. Drug Alcohol Depend.

[19] Bowles JM, Copulsky EC, Reed MK (2024). Media framing xylazine as a “zombie drug” is amplifying stigma onto people who use drugs. Int J Drug Policy.

[20] Chen AT, Wang LC, Johnny S (2025). Stigma and behavior change techniques in substance use recovery: qualitative study of social media narratives. JMIR Form Res.

