# Supplementary material for: Characterization of Reddit Posts About Xylazine-Associated Wounds: Qualitative Study
Source: JMIR Dermatol. 2025 Sep 12;8:e70329. doi: 10.2196/70329 (PMC12434632; doi:10.2196/70329)
Supplement: Multimedia Appendix 1 [file derma-v8-e70329-s001.docx]

**Appendix 1. List of subreddits searched.**

r/xylazine, r/researchchemicals, r/crystalmethanonymous, r/mentalhealthuk, r/offmychest, r/flurazepam, r/dissociatives, r/syntheticcannabinoids, r/ems, r/oxycodone, r/advice, r/growyourownmedicine, r/residency, r/heroinheroines, r/alcohol, r/edmproduction, r/alcoholicsanonymous, r/askscience, r/makeupaddiction, r/DevelopmentalTrauma, r/egames, r/videos, r/REDDITORSINRECOVERY, r/criticaltheory, r/familiesanonymous, r/cfb, r/manga, r/stoicism, r/cyprus, r/buildapc, r/detroithustle, r/callofcthulhu, r/feminism, r/vive, r/casualiama, r/nostupidquestions, r/cosplay, r/martialarts, r/sociology, r/faroeislands, r/shamanism, r/explainlikeimfive, r/rosin, r/London, r/fantasy, r/Bonaire, r/Nicaragua, r/alcoholscience, r/creepy, r/graffiti, r/Canada, r/antiwork, r/saintmartin, r/vinyl, r/darknetmarkets, r/salvia, r/caffeine, r/edm, r/aspergers, r/parkinsons, r/momforaminute, r/benzedrex, r/selfcare, r/therapyabuse, r/cooking, r/anxietyuk, r/migraine, r/guitar, r/eatingdisordersuk, r/stepfamilies, r/Bulgaria, r/chronicpain, r/clobazam, r/relationships, r/law, r/todayilearned, r/artificialintelligence, r/ReagentTesting, r/EDRecovery, r/Monaco, r/youtube, r/risperidone, r/Barbados, r/transgender, r/homebrewdrugs, r/ROCD, r/finland, r/worldnews, r/medicine, r/mensrights, r/streetart, r/health, r/mindfulsobriety, r/abandonedporn, r/anarchism, r/CPTSDpartners, r/DIDpartners, r/soccer, r/quittingkratom, r/pipe, r/starcraft, r/paranoid, r/Misophonia, r/pilates, r/askpsychology, r/lawenforcement, r/sciencefiction, r/existentialism, r/nosleep, r/Drugs, r/ecigs, r/Iceland, r/Jamaica, r/chlordiazepoxide, r/psychonautmarketplace, r/sporetraders, r/medazepam, r/narcoticsanonymousuk, r/neuroscience, r/schizophreniauk, r/atheism, r/pillreports, r/Norway, r/music, r/hydrocodone, r/salviadivinorum, r/dip, r/sanpedrocactusforsale, r/AlcoholicsAnonymous, r/globaldrugsurvey, r/Denmark, r/weightlifting, r/hemp, r/childhoodtrauma, r/benzoscience, r/college, r/mealprep, r/publichealth, r/boxing, r/speedrun, r/homeless, r/filmmakers, r/preppers, r/playstation, r/bipolarSOs, r/saintvincent, r/pregnancy, r/Ativan, r/meperidine, r/adultchildrenofalcoholics, r/raisedbydysfunction, r/ResearchChemicals, r/morphine, r/oxymorphone, r/levorphanol, r/electronicmusic, r/mildlyinteresting, r/history, r/sextalk, r/psychonautwiki, r/pics, r/silkroad, r/police, r/Buddhism, r/hardstyle, r/MaladaptiveDreaming, r/narcoticsanonymous, r/Judaism, r/panama, r/mindfulness, r/wearethemusicmakers, r/anime, r/Seroquel, r/bathsalts, r/Netherlands, r/drums, r/mma, r/weed, r/MDMA, r/TrueOffMyChest, r/treesgrowing, r/cptsd_uk, r/earlypsychosis, r/news, r/Romania, r/CPTSD, r/nonzeroday, r/science, r/AskReddit, r/legalizeitrees, r/Ireland, r/business, r/chronicillness, r/gamingpc, r/shrooms, r/tardivedyskinesia, r/travel, r/motivation, r/peptalks, r/atheistrecovery, r/supervisedinjection, r/libertarian, r/financialindependence, r/Croatia, r/Opiates, r/relationship_advice, r/dominica, r/raisedbynarcissists, r/urbanhell, r/progressnotperfection, r/pots, r/Bahamas, r/EatingDisorders, r/addiction, r/Stoicism, r/selfhelp, r/soberdating, r/nottheonion, r/alandislands, r/teachers, r/caregiversupport, r/mentalhealth, r/legaladvice, r/dancesafe, r/Mescaline, r/Ecuador, r/transgenderrehab, r/rcsources, r/tabletopgames, r/Honduras, r/israel, r/newparents, r/delta8, r/opiateschurch, r/privacy, r/domesticviolence, r/magnesium, r/delta9, r/highdeas, r/twitch, r/Peyote, r/Belgium, r/trees, r/triazolam, r/nursing, r/overemployed, r/art, r/ResponsibleDrugUse, r/socialwork, r/cassetteculture, r/declutter, r/abilify, r/Dissociation, r/tattoo, r/natureisfuckinglit, r/television, r/Albania, r/protectandserve, r/grenada, r/BodyDysmorphia, r/workfromhome, r/Italy, r/bitcoin, r/healthanxietyuk, r/harmreductionsupplies, r/Mexico, r/happy, r/euroents, r/socialism, r/hungary, r/horror, r/mlb, r/microdosing, r/programming, r/foodporn, r/probation, r/altcannabinoids, r/agnosticism, r/askuk, r/naltrexone, r/quazepam, r/hash, r/psychology, r/nba, r/clinicaltrials, r/legalhighs, r/tramadol, r/cryptomarkets, r/roastme, r/addictionuk, r/Xanax, r/AmItheAsshole, r/agingparents, r/ritalin_adults, r/cpr, r/researchchemical, r/rant, r/cscareerquestions, r/stopsmoking, r/cannabiscoupons, r/Austria, r/highstrangeness, r/nicotinepouch, r/alateen, r/incels, r/Prozac, r/femalefashionadvice, r/newzealand, r/running, r/travelEurope, r/skincareaddiction, r/peru, r/bassguitar, r/eupersonalfinance, r/popheads, r/jazz, r/cultivationlinks, r/pansexual, r/sysadmin, r/oxazepam, r/DissociationSupport, r/addictionmedicine, r/leagueoflegends, r/podcasts, r/SuicideWatch. r/benzogirls, r/opiaterollcall, r/soberparenting, r/sex, r/preschoolers, r/redditorsinrecoveryuk, r/romancebooks, r/griefsupport, r/gradschool, r/recipes, r/meditationuk, r/bodyacceptance, r/narcissisticabuse, r/burningman, r/publichealthpolicy, r/valorant, r/alzheimers, r/therapyuk, r/cocaine, r/gaming, r/esports, r/askengineers, r/oddlysatisfying, r/prohibitionends, r/InternalFamilySystems, r/entrepreneur, r/Opioid_RCs, r/ComplexTrauma, r/buprenorphine, r/harmreductionuk, r/classicalmusic, r/Luxembourg, r/GetMotivated, r/opiatesrecovery, r/audioengineering, r/akathisia, r/seedswap, r/punk, r/awakened, r/family, r/anime_irl, r/turkey, r/bushcraft, r/Lexapro, r/Russia, r/collapse, r/psychiatryuk, r/askeurope, r/secularsobriety, r/hiphopheads, r/endometriosis, r/portlandhobos, r/codependency, r/halazepam, r/damnthatsinteresting, r/SaferPartyPractices, r/diazepam, r/EOOD, r/Wellbutrin, r/firstaid, r/astralprojection, r/empathogens, r/depressionuk, r/justnofamily, r/wholesomememes, r/books, r/pharmacology, r/lean, r/wales, r/cryptocurrencies, r/epidemiology, r/Andorra, r/hardcore, r/alcoholicsanonymousuk, r/saintbarthelemy, r/learnprogramming, r/Haiti, r/Australia, r/opiatescience, r/linguistics, r/Switzerland, r/invisibleillness, r/youngadultbooks, r/Liechtenstein, r/movies_irl, r/backpacking, r/yoga, r/glitchinthematrix, r/heroin, r/jersey, r/india, r/Bolivia, r/therapy, r/BorderlinePDisorder, r/Existential_Crisis, r/swimming, r/music_irl, r/personalfinanceuk, r/refugerecoveryuk, r/leaves, r/Dexedrine, r/musicians, r/schizophreniaSOs, r/augmentedreality, r/alanonuk, r/scifi, r/adhdwomen, r/heroinanonymous, r/emotionalneglect, r/kindvoice, r/retrogaming, r/unpopularopinion, r/technology, r/defi, r/countrymusic, r/alcoholism, r/cbd, r/depressants, r/ibs, r/piercing, r/Bermuda, r/philosophy, r/getdisciplined, r/Vyvanse, r/hiking, r/audiophile, r/oculus, r/adultchildrenuk, r/breakups, r/drugpolicy, r/ketamine, r/stopbenzos, r/minimalism, r/art_irl, r/psytrance, r/rapecounseling, r/asexuality, r/piano, r/womeninrecovery, r/SurvivorsSupport, r/legalizemarijuana, r/disneyplus, r/metal, r/AMA, r/fibromyalgia, r/eatcheapandhealthy, r/Japan, r/psychonaut, r/costarica, r/hearthstone, r/formula1, r/quittingsmokingcanada, r/nfl, r/naranon, r/waxpenuk, r/methadone, r/Greece, r/bipolarreddit, r/gifs, r/synthesizers, r/sobermotivation, r/rock, r/estazolam, r/cracksmokers, r/dabs, r/Stims, r/spacex, r/PanicAttack, r/chewingtobacco, r/crohnsdisease, r/careerguidance, r/tv_shows_irl,r/stimscience, r/Effexor, r/movies, r/CompulsiveSkinPicking, r/valium, r/concerta, r/studentloans, r/medicaladvice, r/decriminalize, r/brasil, r/melatonin, r/nutrition, r/liberal, r/singularity, r/astronomy, r/ObscureDrugs, r/DrugInfo, r/smartrecoveryr, r/divorce, r/treatmentforaddiction, r/justnomil, r/networking, r/steamvr, r/speedballs, r/electronic_cigarette, r/anxiety, r/xboxone, r/marijuanaanonymous, r/sobriety_milestones, r/spiritmolecule, r/psychedelicscience, r/meninrecovery, r/hulu, r/anthropology, r/meditation, r/internetparents, r/solotravel, r/soberliving, r/frugalmalefashion, r/refugerecoveryr, r/askphilosophy, r/golf, r/Montenegro, r/trance, r/Paxlovid, r/politics, r/history_irl, r/wallstreetbets, r/cbb, r/isleofman, r/Christianity, r/investing, r/stocks, r/needleexchange, r/changemyview, r/lupus, r/smartrecoveryuk, r/homelessness, r/smokeless, r/beyondthebump, r/naranonuk, r/liminalspace, r/Depersonalization, r/roadtrips, r/Venezuela, r/alcoholism_medication, r/nasa, r/czechrepublic, r/el, r/addictedtotheneedle, r/manga_irl, r/benzodiazepines, r/3dprinting, r/LSD, r/cuba, r/confessions, r/rpgdesign, r/housemusic, r/alprazolam, r/DPDR, r/personalfinance, r/Sweden, r/microgrowerycanada, r/OCDproblems, r/drugresearch, r/askhistorians, r/deliriants, r/adderallstories, r/prazepam, r/selfimprovementuk, r/vapeporn, r/theanine, r/indiangaming, r/frugal_jerk, r/self, r/lorazepam, r/learnjavascript, r/learnmachinelearning, r/backrooms, r/benzowithdrawal, r/webdev, r/onedayatatime, r/dxm, r/rehabandrecovery, r/personalfinancecanada, r/personaldevelopment, r/focalin, r/polyamory, r/opiateswithdrawal, r/psychonaut101, r/Germany, r/funny, r/Greenland, r/pokemontcg, r/cybersecurity, r/Poland, r/AddictionAdvice, r/entwives, r/lithium, r/raspberrypi, r/nbome, r/tilidine,r/flunitrazepam, r/sober, r/mechanicalkeyboards, r/abandonedphotography, r/adultsurvivors, r/dnd, r/space, r/poverty, r/Macedonia, r/saplings, r/dreams, r/rollacigarettes, r/technology_irl, r/chamomile, r/chemistry, r/temazepam, r/biology, r/DecidingToBeBetter, r/schizoaffective, r/hydromorphone, r/Lamictal, r/cycling, r/womensrights, r/curacao, r/cannaverse, r/meth, r/nintendoswitch, r/askdrugs, r/compsci, r/nutmeg, r/doomers, r/booksuggestions, r/2cb, r/tennis, r/natureismetal, r/DNMAvengers, r/Portugal, r/stopsnuffing, r/addictionprevention, r/oilpen, r/urbanplanning, r/zolpidem, r/supplements, r/dumpsterdiving, r/hobo, r/anticonsumption, r/korea, r/yugioh, r/Depakote, r/soberhousing, r/toddlers, r/southafrica, r/gabapentin, r/ptsdpartners, r/belize, r/Colombia, r/ejuice, r/dryalcoholics, r/antiguaandbarbuda, r/ADHD, r/robotics, r/blockchain, r/puertorico, r/rationalpsychonaut, r/psychoticexperiences, r/Zoloft, r/paramedics, r/baking, r/Ritalin, r/disability, r/schizophrenia, r/dementia, r/unitedkingdom, r/startups, r/warhammer40k, r/aviation, r/ptsd_uk, r/Trichotillomania, r/nicotine, r/lifehacks, r/indie, r/Hoarding, r/ocduk, r/ptsd, r/depressionregimens, r/virtualreality, r/tapentadol, r/entactogens, r/Scrupulosity, r/unsolicitedadvice, r/waxpen, r/nitrous, r/Slovakia, r/techsupport, r/fire, r/animalsbeingbros, r/simpleliving, r/codeine, r/gadgets, r/abusiverelationships, r/bartardstories, r/DID, r/Islam, r/singing, r/psychiatry, r/Slovenia, r/virginislands, r/smartrecovery, r/eucigs, r/clonazepam, r/exmormon, r/socialjustice101, r/France, r/recoveringredditors, r/vagabond, r/lesbian, r/Cymbalta, r/lormetazepam, r/consolemasterrace, r/conservative, r/screenwriting, r/eszopiclone, r/haircare, r/Arduino, r/dubstep, r/dominicanrepublic, r/elementaryteachers, r/kratom, r/EmotionalSobriety, r/Argentina, r/legaladviceofftopic, r/testkits, r/harmreduction, r/DrugNerds, r/headphones, r/motogp, r/popping, r/biohackers, r/mengetwell, r/bromazepam, r/sexualassault, r/Recovery, r/loseit, r/sexpositive, r/progresspics, r/spiritualpractice, r/science_irl, r/infertility, r/careeradvice, r/aruba, r/estrangedadultchildren, r/raisedbyborderlines, r/cannabisextracts, r/apexlegends, r/AskDrugNerds, r/cancer, r/productivity, r/bodyart, r/diy, r/dihydrocodeine, r/tooafraidtoask, r/saintlucia, r/HarmOCD, r/ketazolam, r/cbdoil, r/DissociativeIdentity, r/china, r/hockey, r/Anhedonia, r/boardgames, r/askdoctors, r/klonopin, r/valerianroot, r/thesoberlife, r/Netflix, r/drugpolicyuk, r/Serbia, r/spacebuckets, r/gaming_irl, r/mademesmile, r/whatshouldiread, r/fentanyl, r/casualconversation, r/heroinrecovery, r/pcgaming, r/powerlifting, r/minipainting, r/tetrazepam, r/sanmarino, r/vaporentscanada, r/spain, r/hallucinations, r/sobercurious, r/opiates, r/autism, r/magicthegathering, r/suboxone, r/aves, r/relationshipadvice, r/engineering, r/dph, r/OCD, r/festivals, r/alanon, r/PsychedSubstance, r/benzorecovery, r/primevideo, r/electronics, r/luciddreaming, r/HealthAnxiety, r/Egypt, r/urbanexploration, r/guernsey, r/overwatch, r/cigar, r/Narcan, r/vaticancity, r/camping, r/goa, r/photography, r/Hinduism, r/economics, r/aromantic, r/pathfinder_rpg, r/AdultChildren, r/nihilism, r/lyrica, r/gay, r/bisexual, r/propoxyphene, r/ultrawidemasterrace, r/bosnia, r/stonerengineering, r/spirituality, r/buddhism, r/Kosovo,r/malta, r/ausents, r/microgrowery, r/kief, r/cleanandsober, r/streetphotography, r/Scotland, r/pethidine, r/TripSit, r/ulcerativecolitis, r/womeninsobriety, r/decidingtobebetter, r/pcos, r/toxicparents, r/quittingweed, r/vaporents, r/books_irl, r/opiates_gonewild, r/homelab, r/frugal, r/ashwagandha, r/meditationtechniques, r/refugerecovery, r/altcoins, r/toastme, r/selfimprovement, r/friendship, r/bipolaruk, r/aww, r/askmeanything, r/zaleplon, r/OpiatesRecovery, r/Ethereum, r/Gibraltar, r/nitrazepam, r/olanzapine, r/stopdrinking, r/Suboxone, r/dating_advice, r/kava, r/overdoseprevention, r/bjj, r/blues, r/communism, r/Adderall, r/midazolam, r/cannabinoidscience, r/Ayahuasca, r/erowid, r/Motivation, r/zopiclone, r/magicplantsexchange, r/seattlehobos, r/sintmaarten, r/depression, r/ehlersdanlos, r/folk, r/dota2, r/socialanxiety, r/learnpython, r/futurology, r/nootropics, r/graphicnovels, r/UpliftingNews, r/fitness, r/vent, r/Guatemala, r/DMT, r/flakka, r/clozapine, r/dadforaminute, r/trinidadandtobago, r/uktrees, r/delusions, r/exchristian, r/lgbtqsobriety, r/swingers, r/drugdetox, r/flightsim, r/jobs, r/lgbtq_plus, r/acting, r/cryptocurrency, r/Europe, r/conspiracy, r/munchies, r/codauk, r/corrections, r/Taoism, r/Palestine, r/eyebleach, r/comics_irl, r/bodybuilding, r/SanPedroCactus, r/exjw, r/PureOCD, r/fortnitebr, r/survival, r/offgrid, r/animalsbeingderps, r/multiplesclerosis, r/comics, r/cocaineanonymous, r/psychic, r/politicalscience, r/videography

**Appendix 2. Drug and wound-related terms and lexical variants searched.**

Drug related terms

xylazine

tranq

zombie drug

sleep-cut

xilazine

xlyazine

xylasine

xylazene

xylazime

xylazne

xylzene

xylzine

zalazine

zylasine

zylazene

zylazine

zylozine

Wound related terms:

Abscess

amputation

cellulitis

flesh

infection

lesion

necrosis

skin

swollen

tissue

ulcer

wound

sore

**Table S1. Theme definitions.**

| Theme | Description | Letter code | Posts coded |
| --- | --- | --- | --- |
| Xylazine Use Habits | Posts where people identify how they personally use xylazine, injection vs snort (should exclude people talking about how other people use) | A | 19 |
| Other Drugs use habits | Posts where people mention what other drugs they use, fent, oxy, benzos etc | B | 20 |
| Locations of wounds on the body | Posts that describe where wounds occur on the body, looking for mouth, nose, extremities or other locations | C | 56 |
| Management of Wounds | Posts about what people are doing to manage wounds, dressings, changing, medical interventions like antibiotics, hospital admission, amputations | D | 34 |
| Stigma Related to Xylazine wounds | Posts about stigma, or posts using stigmatizing language, ie zombie, flesh eating, apocalypse | E | 23 |
| Ability to get into rehab clinics | Posts about being able to get into rehab clinics, xylazine wound related or not. | F | 10 |
| Hypothesized mechanism of Xylazine | Posts trying to explain why xylazine does what it does, looking for comparisons to other drugs, like krokodil and clonidine, or alpha effects | G | 84 |
| Posts about specific xylazine withdrawal symptoms | Any posts that mention xylazine withdrawal specifically, would exclude ones that don’t specify substance withdrawing from | H | 29 |
| Posts about MOUDs | Any posts that mention methadone, buprenorphine, subs, etc | I | 19 |
| Non-MOUD management of withdrawal | Posts about the management of xylazine withdrawal symptoms with non-moud medications like gabapentin, benzos, clonidine. | J | 11 |
| Non-relevant post about xylazine | A post that mentions xylazine but doesn’t seem to be about personal use, withdrawal, wounds. | K | 67 |
| Geographic region | Posts that specifically include geographical demographic information. Looking for specific mentions of location and presence of xylazine in the area | L | 37 |
| Not about xylazine at all | A post that has nothing to do with xylazine, wounds, withdrawal | X | 70 |
